# Geriatric care in traditional communities of Central Asia

**DOI:** 10.11604/pamj.2025.52.94.49827

**Published:** 2025-11-03

**Authors:** Aleksandr Martynenko

**Affiliations:** 1 Department of Internal Medicine, LLC “Multifunctional Medical Center” M-clinic, Tashkent, Uzbekistan

**Keywords:** Healthy ageing, geriatrics, traditional societies, family structure, non-communicable diseases, cultural practices, physical activity, community health programs, Central Asia, Africa

## To the editors of the Pan African Medical Journal

Modern geriatrics, considering the approach of the World Health Organization (WHO), focuses on functional ability - the ability of older people to maintain independence and well-being through a combination of medical and social care [1]. This approach combines healthcare that identifies geriatric syndromes with social support tailored to individual needs. In countries such as Uzbekistan (Central Asia), which is in the process of modernization, older people are a unique phenomenon. They are keepers of traditions and memories of generations based on strong family ties, and face challenges related to cultural practices that affect their nutrition, physical activity, and health. Similar patterns are observed in many African societies, where older adults also play a central role in maintaining cultural identity and intergenerational cohesion. We analyze how traditional society shapes aging and consider socially integrated solutions to overcome existing barriers.

The population of the country is 37.5 million people: young people (the average age is 29.25 years), but 2 million over 65, are becoming an increasingly visible group [2]. These elderly people live in multigenerational families, which ensures a high level of socialization. Elders are highly respected (kattalarga khurmat), which makes them key figures: their opinion is valued in making decisions and household holding. A comparable situation exists in many African communities, where respect for elders and collective living traditions remain cornerstones of social structure.

However, this structure has a downside. Living in large families deprives one of personal space and autonomy. Women cook pilaf that takes a long time, and men work in the garden or graze cattle in rural areas. Of course, joint work strengthens connections, but constant noise and congestion increase stress, which leads to deterioration in the course of arterial hypertension and diabetes. Similar challenges have been reported in African households, where shared spaces and communal responsibilities, while strengthening bonds, can also limit individual autonomy and contribute to stress-related health outcomes.

Relatives often arrange a kind of competition: who can provide the best care for elderly family members, which sometimes leads to overtreatment and even disability. This form of family-based “protective care”, although rooted in respect and tradition, is also recognizable in African settings, where strong family responsibility can sometimes unintentionally lead to overprotection and delayed professional medical care.

Nutrition and physical activity highlight the conflict between traditions and health. Uzbek cuisine (shish kebab, samsa, and pilaf) is rich in fats and meat; it dominates family feasts (gap or hashtak), where the elderly actively participate. Vegetables and fruits, despite their availability, are eaten little. This nutritional pattern parallels dietary habits in several African regions, where traditional celebrations also often center on calorie-dense meals, contributing to rising burdens of non-communicable diseases. The data confirm the consequences: 21.8% of adult (aged 18 years and over) women and 16.1% of adult men are living with obesity, and in Tashkent over the past five years, the prevalence of obesity has increased more than 6 times [3]. Almost every third patient seeking help has impaired fasting glycemia or type 2 diabetes, and arterial hypertension is not uncommon even among young people. Low physical activity makes the situation worse. The main activity of the elderly is limited to household chores, which doesn´t compensate for the risk of osteoporosis, arthritis, or cardiovascular diseases [4,5]. Likewise, low levels of structured physical activity among older adults are a recognized public health challenge in both Central Asian and African traditional societies.

The elderly in Uzbekistan are not consciously isolated, but their attitude to their health is limited by traditions - a paradox of caring and dependence. Overprotection from children, illness as an excuse for increased attention - all this is a part of the life of the local traditional population, which has been formed over the centuries. This paradox is also present in many African communities, where older adults are simultaneously deeply integrated into family life yet have limited agency in making health-related decisions.

Community-based programs could be a solution. Volunteers or trained relatives can explain to families the principles of managing chronic diseases, promote a balanced diet and light activities, such as walking with family, which corresponds to cultural values. Clinics can involve volunteers in the schools of healthy aging, aimed at educating various segments of the population on the basics of healthy longevity.

## Conclusion

The elderly of Uzbekistan reflect a global challenge: the harmonization of traditions and healthy aging. Their family-based lives, but complicated by unhealthy diets, physical inactivity, and often outdated treatment approaches, provide lessons specific to geriatric care for traditional societies. This experience can be valuable not only for Central Asia but also for African regions facing similar demographic transitions and cultural dynamics. The integration of medical, social, and community strategies can transform the elderly population not only to survive, but to thrive within their traditions [6].
